# Approximating Vector Scheduling: Almost Matching Upper and Lower Bounds

**DOI:** 10.1007/s00453-016-0116-0

**Published:** 2016-01-19

**Authors:** Nikhil Bansal, Tim Oosterwijk, Tjark Vredeveld, Ruben van der Zwaan

**Affiliations:** 1 Eindhoven University of Technology, Eindhoven, The Netherlands; 2 Maastricht University, Maastricht, The Netherlands

**Keywords:** Integer Linear Program, Multiprocessor Schedule, Resource Augmentation, Optimum Integer Solution, Vector Schedule

## Abstract

We consider the Vector Scheduling problem, a natural generalization of the classical makespan minimization problem to multiple resources. Here, we are given *n* jobs, represented as *d*-dimensional vectors in $[0,1]^d$, and *m* identical machines, and the goal is to assign the jobs to machines such that the maximum *load* of each machine over all the coordinates is at most 1. For fixed *d*, the problem admits an approximation scheme, and the best known running time is $n^{f(\epsilon ,d)}$ where $f(\epsilon ,d) = (1/\epsilon )^{\tilde{O}(d)}$ ($\tilde{O}$ suppresses polylogarithmic terms in *d*). In particular, the dependence on *d* is double exponential. In this paper we show that a double exponential dependence on *d* is necessary, and give an improved algorithm with essentially optimal running time. Specifically, we let $\exp (x)$ denote $2^x$ and show that: (1) For any $\epsilon <1$, there is no $(1+\epsilon )$-approximation with running time $\exp \left( o(\lfloor 1/\epsilon \rfloor ^{d/3})\right)$ unless the Exponential Time Hypothesis fails. (2) No $(1+\epsilon )$-approximation with running time $\exp \left( \lfloor 1/\epsilon \rfloor ^{o(d)}\right)$ exists, unless NP has subexponential time algorithms. (3) Similar lower bounds also hold even if $\epsilon m$ extra machines are allowed (i.e. with resource augmentation), for sufficiently small $\epsilon >0$. (4) We complement these lower bounds with a $(1+\epsilon )$-approximation that runs in time $\exp \left( (1/\epsilon )^{O(d \log \log d)}\right) + nd$. This gives the first efficient approximation scheme (EPTAS) for the problem.

## Introduction

We consider the Vector Scheduling problem defined as follows. The input consists of a collection *J* of *n* jobs $\mathbf {p_1},\ldots ,\mathbf {p_n}$, viewed as *d*-dimensional vectors from $[0,1]^d$, and *m* identical machines. The goal is to find an assignment of the jobs to the machines such that the load satisfies $\left\| \sum _{\mathbf {p} \in P_i} \mathbf {p}\right\| _\infty \le 1$ for each machine $i \in [m]$, where $P_i$ is the set of jobs assigned to machine *i*. That is, the maximum load on any machine in any coordinate is at most 1.

Vector Scheduling is the natural multi-dimensional generalization of the classic Multiprocessor Scheduling problem (also known as makespan minimization, $P||C_{\max }$, or load balancing). In the latter problem, the goal is to assign *n* jobs with arbitrary processing times to *m* machines in order to minimize the maximum sum of processing times (load) over all the machines. However, for many applications, the jobs may use different resources and the load of a job cannot be described by a single aggregate measure. For example, if jobs have both CPU and memory requirements, their processing requirement is best modeled as a two-dimensional vector, where the value in each coordinate corresponds to each of the requirements. Note that the assumption that the maximum load of a machine in any coordinate is 1 is without loss of generality, as the different coordinates can be scaled independently.

In this paper we are concerned with approximation algorithms. We say that an algorithm is an $\alpha$-approximation for some $\alpha > 1$ if it finds an assignment with load at most $\alpha$, whenever there exists a feasible schedule with load at most 1.

### Previous Work

Multiprocessor Scheduling and the related Bin Packing problem are two of the most fundamental problems in combinatorial optimization with a long and rich history. We only describe the work on Multiprocessor Scheduling in the setting where the number of machines *m* is part of the input. It is well-known that Multiprocessor Scheduling is strongly NP-hard [10].

The first polynomial time approximation scheme (PTAS), that is, a $(1+\epsilon )$-approximation algorithm with polynomial running time for every fixed $\epsilon >0$, was obtained by Hochbaum and Shmoys [11]. The running time of their algorithm is $O\left( n^{O(1/\epsilon ^2)}\right)$. Note that by the strong NP-Hardness of the problem one cannot hope to have a running time with polynomial dependence in $\epsilon$ (i.e. an FPTAS), unless P = NP.

An efficient polynomial time approximation scheme (EPTAS), i.e. an algorithm with running time $f(\epsilon )n^{O(1)}$, was implicit in [11] by replacing the dynamic program by an integer linear program and using fast integer programming algorithms in fixed dimensions. Alon et al. [1] developed a more general framework to obtain EPTASes for parallel machine scheduling that runs in $f(\epsilon ) + O(n)$ time, where $f(\epsilon )$ is a double exponential function in $1/\epsilon$.

Recently, this running time was substantially improved by Jansen [14] to $O\left( 2^{\tilde{O}(1/\epsilon ^2)} + n^{O(1)}\right)$. His main idea is to use fast integer programming in fixed dimensions, together with an elegant result of Eisenbrand and Shmonin [6] about the existence of optimum integer solutions with small support. Most of these results also extend to the setting of uniform machines, i.e. a setting where the machine speeds differ (see e.g. [12, 14]).

Fewer results are known for the case when the number of dimensions exceeds one. Chekuri and Khanna [5] gave the first polynomial-time approximation scheme for a fixed number of dimensions. They gave an algorithm with running time $n^{g(\epsilon ,d)}$, where $g(\epsilon ,d) = (1/\epsilon )^{d\log \log d + o(d)}$ and hence the running time is $n^{(1/\epsilon )^{\tilde{O}(d)}}$. This seems to be the currently best known running time for this problem. PTASes for several other generalizations are also known [3, 7, 8].

When *d* is part of the input, Chekuri and Khanna [5] gave a polynomial time $O(\ln ^2 d)$-approximation and proved that it is NP-hard to approximate the problem within any constant factor. This approximation factor has been recently improved to $O(\log d)$ by Meyerson et al. [18]. The latter result even holds in the online setting.

### Our Contribution

A natural question is whether there exists an approximation scheme for Vector Scheduling with a single exponential running time in $1/\epsilon$ and *d*, e.g. $\exp ({\text {poly}(1/\epsilon ,d)})$. We rule out this possibility by showing the following strong lower bound.

#### **Theorem 1**

For any $\epsilon > 0$ with $1/\epsilon \in \mathbb {N}$, there is a $d(\epsilon )$ such that there is no $(1+\epsilon )$-approximation algorithm with running time $O\left( 2^{o\left( (1/\epsilon ) ^{d/3}\right) } (nd)^{O(1)} \right)$ for Vector Scheduling in $d\ge d(\epsilon )$ dimensions, unless the Exponential Time Hypothesis (ETH) fails.

This follows from a relatively simple reduction from the 3-Dimensional Matching problem. The same reduction also implies the following hardness under a more standard complexity assumption.

#### **Theorem 2**

For any $\epsilon > 0$ with $1/\epsilon \in \mathbb {N}$, there is a $d(\epsilon )$ such that there is no $(1+\epsilon )$-approximation algorithm with running time $O\left( 2^{(1/\epsilon )^{o(d)}} (nd)^{O(1)}\right)$ for Vector Scheduling in $d\ge d(\epsilon )$ dimensions, unless NP has subexponential time algorithms, i.e. NP $\subseteq \cap _{\epsilon >0} \text {DTIME}(2^{n^\epsilon })$.

One may wonder whether these lower bounds are robust or whether they crucially exploit the fact that no additional machines are allowed. It is instructive to consider the case of $d=1$ (i.e. Multiprocessor Scheduling). Recall that no FPTAS is possible for the problem. However, if one allows some extra machines (say $\lceil \epsilon m \rceil$ of them), then the running time dependence on $\epsilon$ reduces dramatically and in particular, an FPTAS is possible. In fact, the known FPTASes for Bin Packing imply that even very few extra machines (poly-logarithmic in *m*) suffice [16, 20], and in fact one does not even need to violate the capacity of any machine.

Somewhat surprisingly, we show that extra machines do not help for Vector scheduling, provided that the desired approximation ratio is sufficiently small.

#### **Theorem 3**

For any $\epsilon < \epsilon _0$ with $1/\epsilon \in \mathbb {N}$, there is a $d(\epsilon )$ such that there is no $(1+\epsilon )$-approximation algorithm with running time $O\left( 2^{(1/\epsilon )^{o(d)}} (nd)^{O(1)}\right)$ for Vector Scheduling in $d\ge d(\epsilon )$ dimensions, even with $\lceil \epsilon m \rceil$ extra machines, unless NP has subexponential time algorithms, i.e. NP $\subseteq \cap _{\epsilon >0} \text {DTIME}(2^{n^\epsilon })$, where $\epsilon _0 < 1$ is a universal constant. Assuming the ETH, no such algorithm can run in time $O\left( 2^{o\left( (1/\epsilon )^{d/6}\right) } (nd)^{O(1)}\right)$.

To complement the lower bounds above, we show the following algorithmic result.

#### **Theorem 4**

For any $\epsilon >0$ and $d \ge 1$, there is a deterministic $(1+\epsilon )$-approximation algorithm for *d*-dimensional Vector scheduling that runs in time $O\left( 2^{(1/\epsilon )^{O(d \log \log d)}}+ nd \right) .$

By the lower bounds above, the running time is essentially the best possible (modulo the $O(\log \log d)$ factor in the exponent), and the *nd* term is simply the time required to read the input. Theorem 4 gives the first EPTAS for Vector Scheduling.

#### Techniques

At a high level, the algorithm is similar to that of [14], and relies on integer programming in fixed dimensions and the existence of optimum integer solutions with small support. However, there are some important differences between $d=1$ and $d>1$. In particular, for $d=1$ the small jobs (with size $\le \epsilon$) do not cause any problems and can later be assigned greedily in the remaining space, after solving the problem for just big jobs. However, for $d\ge 2$, the big and small jobs (by small we mean jobs that are small in *every* dimension) interact in more complex ways and must be considered together. The following example illustrates this difficulty.

##### *Example 1*

Consider the following instance in $d=2$ dimensions, with $m=2$ machines and the following jobs: $\mathbf {p_1} = \left( \frac{1}{2}, 0\right) , \mathbf {p_2} = \left( \frac{1}{2},0\right)$ and $\mathbf {p_i} = \left( \frac{\epsilon }{2}, \epsilon \right) \text { for } 3 \le i \le 2/\epsilon .$ Clearly, these jobs can be scheduled on two machines by assigning the first two jobs to separate machines and splitting the small jobs evenly. However, if the two large jobs are assigned to the same machine, there is no assignment of the small jobs such that the maximum load of the machines is exceeded by a constant factor dependent on $\epsilon$. The two large jobs have total load (1, 0). As the small jobs have total load (1, 2), no matter how these are assigned to the two machines, one machine will have load at least $\min \left\{ \max \{1+x,2x\},\max \{1-x,2(1-x)\}\right\}$, which is 4 / 3 (attained for $x=1/3$).

Chekuri and Khanna [5] overcame this problem by ‘guessing’ the division between small and large jobs for each machine. This allows them to decouple the assignment of small and big vectors. However, as there are roughly $m^{(1/\epsilon )^d}$ different possible divisions, with $\epsilon$ precision, this is not useful to obtain an efficient polynomial time approximation scheme.

To get around this, we incorporate both large and small vectors in our mixed integer linear program ($\mathsf {MILP}$), but ensure that it has only few constraints by tracking only some coarse-grained information for the small jobs. We find an optimum solution to this $\mathsf {MILP}$, which gives an integral assignment of large jobs, but small jobs might be assigned fractionally. We then show how to assign the small jobs to machines without overloading them. To do this, we first assign the jobs greedily guided by a potential function, which guarantees that the aggregate amount of overload on machines is small. This load is small enough to ensure that the jobs on overloaded machines can be redistributed in a round-robin manner. A naive implementation of the greedy assignment requires *O*(*mn*) time (as for each job, we need to determine which machine causes the least increase in potential), so we also present some additional ideas to show how everything can be done in linear time.

#### Organization

In Sect. 2 we state our notation and the hypotheses on which our lower bounds are based, and we describe the relevant background on integer programming. In Sect. 3 we prove our lower bounds for Vector Scheduling and we present our algorithm in Sect. 4.

## Preliminaries

Let [*n*] denote the set of positive integers 1 to *n*, i.e. $[n] := \{1,\ldots ,n\}$. Let $\mathbf {1}$ be the all-ones vector. For a *d*-dimensional vector $\mathbf {v}=(v_1,\ldots ,v_d)$, let $v_j$ denote its *j*-th coordinate. For two vectors $\mathbf {a},\mathbf {b}$ we say that $\mathbf {a} \le \mathbf {b}$ if $a_i \le b_i$ for all *i*. Throughout the paper the logarithm $\log$ is taken with base 2 and we let $\exp (x)$ denote $2^x$. We say that a function *f*(*n*) is sub-exponential if $f(n) \in O\left( 2^{O(n^{o(1)})}\right)$. Without loss of generality we assume that the number of machines is less than the number of jobs (otherwise assign one job per machine or conclude infeasibility).

In the 3-CNF-Sat problem, we are given a Boolean expression in conjunctive normal form, consisting of *N* variables and *M* clauses that each consist of three literals. The question is whether or not there exists an assignment of logical values to the variables such that the expression evaluates to TRUE. Impagliazzo, Paturi and Zane formulated the Exponential Time Hypothesis, which in combination with the sparsification lemma [4] can be stated as follows.

### **Hypothesis 1**

(*Exponential Time Hypothesis (ETH)* [13]) 3-CNF-Sat with *N* variables and *M* clauses cannot be solved in time $O\left( 2^{o(M)} (N + M)^{O(1)}\right)$.

We will use the following well-known result for fast integer linear programs with few integer variables.

### **Theorem 5**

(Lenstra [17], Kannan [15], Frank and Tardos [9]) Consider a mixed-integer linear program $\min \{ \mathbf {c}^T \mathbf {x} \ | \ A\mathbf {x} \ge \mathbf {b} \text { and } \forall i \in \mathcal {I}:x_i \in \mathbb {Z} \}$ with *n* variables and *m* constraints, and where $\mathcal {I} \subseteq [n]$ denotes the set of indices of integer variables. Let *s* denote the binary encoding length of the input. There is an algorithm that finds a feasible solution or decides that there is no feasible solution in $O\left( n^{2.5n+o(n)} \cdot s\right)$ arithmetic operations.

Relatively recently, based on an elegant pigeonhole argument, Eisenbrand and Shmonin [6] showed that every feasible integer linear program has an optimum solution with small support.

### **Theorem 6**

(Eisenbrand and Shmonin [6]) Let $\min \{\mathbf {c}^T \mathbf {y} |A \mathbf {y} = \mathbf {b}, \mathbf {y} \ge 0, \mathbf {y} \in \mathbb {Z}^n\}$ be an integer program, where $A \in \mathbb {Z}^{m\times n}$ and $\mathbf {c} \in \mathbb {Z}^n$. If this integer program has a finite optimum, then there exists an optimal solution $\mathbf {y^*}\in \mathbb {Z}^n_{\ge 0}$ in which the number of nonzero components is at most $2(m +1)(\log (m +1)+s+2)$, where *s* is the largest size in binary representation of any coefficient of *A* and $\mathbf {c}$.

## Lower Bounds on the Running Time

We prove our lower bounds by a reduction from 3-Dimensional Matching (3-DM) to Vector Scheduling. In Sect. 3.1 we prove Theorem 1 by describing the reduction and proving that an approximate solution to the Vector Scheduling instance implies an exact solution for 3-DM and hence 3-CNF-Sat. In Sect. 3.2 we outline how the same reduction implies Theorem 2. Finally, in Sect. 3.3 we give the proof for Theorem 3 concerning resource augmentation.

Before we give our reduction, we first define the 3-Dimensional Matching problem. An instance of 3-DM consists of three disjoint sets *X*, *Y*, and *Z*, satisfying $|X| = |Y| = |Z| := q$, and a set $T \subset X \times Y \times Z$ of triples. The goal is to find a subset of triples $T' \subset T$ such that each element of *X*, *Y*, and *Z* occurs in exactly one triple of $T'$.

In [10], a reduction from 3-CNF-Sat to 3-DM is given, that transforms instances of 3-CNF-SAT with *N* variables and *M* clauses into instances for 3-DM with $q = 6M$ and $|T| = 2MN + 3M + 2M^2N(N-1)$ (using better bookkeeping you can prove that $|T|=17M$ suffices). Therefore, the ETH (Hypothesis 1) implies there is no $O(2^{o(q)} |T|^{O(1)})$ time algorithm for 3-DM.

### Lower Bound Assuming the ETH

#### The Construction

The main idea of the reduction is the following construction of a Vector Scheduling instance from 3-DM. For each triple in *T* we construct a job (that we call a *triple-job*), and for each element in *X*, *Y* or *Z* we construct as many jobs as the number of times this element occurs in the triples (we call such jobs *element-jobs*). We explicitly refer to *X-jobs, Y-jobs and Z-jobs* if we want to distinguish the element-jobs of the three sets. For each element *i*, we designate exactly one of its jobs as the *real* element-job corresponding to *i*, and refer to the other element-jobs of *i* as *dummy* jobs. The number of machines is equal to the number of triples. We will assign sizes to these jobs such that to obtain a schedule where the maximum load in any coordinate is at most 1, we need to schedule each triple together with its corresponding three element-jobs, and moreover these element-jobs are either all *real* or all dummy element-jobs.

Let $\epsilon > 0$ be such that $1/\epsilon$ is integer. Let $b = 1/\epsilon - 1$ and let $\mathbf {b}$ denote the vector that has *b* in every coordinate. By $\langle i\rangle$ we denote the ($b+1$)-ary encoding of the integer *i* and by $\overline{\langle i\rangle }$ we denote its complement, that is, $\overline{\langle i\rangle } := \mathbf {b} - \langle i\rangle$. Let $\langle i\rangle _j$ denote the *j*-th digit from the right of $\langle i\rangle$. For ease of notation, we scale the jobs by a factor *b*. That is, all jobs are vectors in $\{0,\ldots ,b\}^d$ and we want to know whether we can schedule the jobs such that the maximum load in each coordinate is at most *b*. To make the proofs easier to read, we rename the elements in the sets *X*, *Y* and *Z* by assuming that $X = Y = Z = \{1,\ldots ,q\}$.

#### The Formal Reduction

Given an instance (*X*, *Y*, *Z*; *T*) of 3-DM, let $n_X(i)$ denote the number of triples (*x*, *y*, *z*) for which $x = i$; in a similar way, we define $n_Y(i)$ and $n_Z(i)$. For each element $i \in X$, we create $n_X(i)$ jobs, one *real* *X*-job *i* and $n_X(i)-1$ *dummy* *X*-jobs. In a similar way, we create $n_Y(j)$ *Y*-jobs for each element $j \in Y$ and $n_Z(k)$ *Z*-jobs for each element $k \in Z$. Finally, we have |*T*| triple-jobs, one for each triple $l \in T$. The number of machines is equal to $m:=|T|$. Note that the total number of jobs is $\sum _{i \in X} n_X(i) + \sum _{j \in Y} n_Y(j) + \sum _{k \in Z} n_Z(k) + |T| = 4 |T|$.

Recall that $|X|=|Y|=|Z|=q$, and let $\ell := \lceil \log _{(1/\epsilon )}q \rceil$. We associate a vector to each of the jobs as in Table 1. These vectors are *d*-dimensional, where $d := 7 + 3 \ell$. In particular, the first four coordinates of a job indicate whether the job corresponds to an element in *X*, *Y*, *Z* or to a triple in *T*. The following three coordinates encode for each *X*, *Y*, or *Z*-job whether it is a real job or a dummy job. The last part of each job encodes the element to which the job corresponds.

**Table 1:** Construction of the jobs from elements and triples of the 3-DM problem

Job name	Values of the coordinates				
	T/*X* / *Y* / *Z*	Real/dummy	Encoding of element(s)		
Real *X*-job *i*:	0, *b*, 0, 0	*b*, 0, 0	$\langle i\rangle _1,\ldots ,\langle i\rangle _\ell$	$0,\ldots ,0$	$0,\ldots ,0$
Dummy *X*-job *i*:	0, *b*, 0, 0	0, *b*, 0	$\langle i\rangle _1,\ldots ,\langle i\rangle _\ell$	$0,\ldots ,0$	$0,\ldots ,0$
Real *Y*-job *j*:	0, 0, *b*, 0	0, *b*, 0	$0,\ldots ,0$	$\langle j\rangle _1,\ldots ,\langle j\rangle _\ell$	$0,\ldots ,0$
Dummy *Y*-job *j*:	0, 0, *b*, 0	0, 0, *b*	$0,\ldots ,0$	$\langle j\rangle _1,\ldots ,\langle j\rangle _\ell$	$0,\ldots ,0$
Real *Z*-job *k*:	0, 0, 0, *b*	0, 0, *b*	$0,\ldots ,0$	$0,\ldots ,0$	$\langle k\rangle _1,\ldots ,\langle k\rangle _\ell$
Dummy *Z*-job *k*:	0, 0, 0, *b*	*b*, 0, 0	$0,\ldots ,0$	$0,\ldots ,0$	$\langle k\rangle _1,\ldots ,\langle k\rangle _\ell$
Triple (*i*, *j*, *k*):	*b*, 0, 0, 0	0, 0, 0	$\overline{\langle i\rangle }_1,\ldots ,\overline{\langle i\rangle }_\ell$	$\overline{\langle j\rangle }_1,\ldots ,\overline{\langle j\rangle }_\ell$	$\overline{\langle k\rangle }_1,\ldots ,\overline{\langle k\rangle }_\ell$

#### Proof of the Reduction

We now show that the reduction has the desired properties.

##### **Lemma 1**

(Completeness) If the 3-DM instance has a solution, then there exists an assignment of the jobs to the *m* machines such that the load on every machine in each coordinate is at most *b*.

##### *Proof*

Consider the collection $T'$ of disjoint triples that cover *X*, *Y* and *Z*. For each triple $(i,j,k) \in T'$ we assign the corresponding triple-job and the real element-jobs corresponding to *i*, *j* and *k* to a single machine. Clearly, every coordinate on every such machine has load at most *b*. We place each of the remaining triples (*i*, *j*, *k*) on a machine with a dummy job for *i*, for *j* and for *k*. It is easily verified that this is a feasible assignment. $\square$

##### **Lemma 2**

If the Vector Scheduling instance has a solution with load at most $(1+\epsilon )b$, then there is a solution to the corresponding 3-DM instance.

##### *Proof*

Consider any solution with load at most $(1+\epsilon )b$. We begin with various properties of such a solution.

##### *Property 1*

The load is exactly *b* in each coordinate on each machine.

##### *Proof*

The load of each machine is at most $(1+\epsilon )b=b+b/(b+1) < b+1$. As all jobs have integer coordinates, the load of each machine is at most *b*.

Moreover, since $\sum _{i \in X} n_X(i) = \sum _{j \in Y} n_Y(j) = \sum _{k \in Z} n_Z(k) = |T| = m$, observe that the total amount of work in the *i*-th coordinate summed over all jobs is *mb*. As all jobs are scheduled and the load is at most *b*, it is *exactly* *b*. $\square$

##### *Property 2*

Each machine processes exactly one triple-job, one *X*-job, one *Y*-job, and one *Z*-job.

##### *Proof*

This follows immediately from the values in the first four coordinates and the previous property. $\square$

##### *Property 3*

Element-jobs assigned to the same machine are either all *real* jobs or all *dummy* jobs.

##### *Proof*

From Property 1 and the values in the fifth, sixth and seventh coordinate we see that the following three statements are simultaneously true:

1. There is exactly one real *X*-job or dummy *Z*-job (coordinate 5);
2. There is exactly one real *Y*-job or dummy *X*-job (coordinate 6);
3. There is exactly one real *Z*-job or dummy *Y*-job (coordinate 7).

The claim now follows by combining this with the fact that by Property 2 there is exactly one (real or dummy) job of each of the types *X*, *Y* and *Z*. $\square$

##### *Property 4*

If a machine processes the triple-job (*i*, *j*, *k*) and a (real or dummy) element-job *a*, then *a* is equal to *i*, *j* or *k*, depending on whether *a* is an *X*, *Y* or *Z*-job.

##### *Proof*

We only consider the case that *a* is an *X*-element; the other cases are similar. By Properties 1 and 2, we know that $\overline{\langle i\rangle } + \langle a\rangle = \mathbf {b}$. Therefore, $\langle a\rangle = \mathbf {b} - \overline{\langle i\rangle } = \mathbf {b} - (\mathbf {b} - \langle i\rangle ) = \langle i\rangle$ and thus $a = i$. $\square$

If a machine processes three real element-jobs, then by the last property the corresponding three elements form a triple in the 3-DM instance. Let $T'$ consist of all triples corresponding to the triple-jobs that are scheduled together with real elements. Then, the triples in $T'$ have no overlap as there is only one real element-job corresponding to an element. Moreover, $T'$ covers all elements, because all jobs, and therefore also all real element-jobs, need to be scheduled.$\square$

Therefore we have the following lemma.

##### **Lemma 3**

Given an instance of 3-Dimensional Matching with $|X|=|Y|=|Z|=q$, $T \subseteq X \times Y \times Z$, $b \in \mathbb {N}_+$, $b \ge 2$ and $\epsilon = 1/(b+1)$, there is a polynomial time reduction to an instance of Vector Scheduling with 4|*T*| vectors in dimension $d := 3\left\lceil \log _{(1/\epsilon )} q\right\rceil + 7$. Moreover, a $(1+\epsilon )$-approximate solution to the Vector Scheduling instance defines a solution to the 3-DM problem.

Thus, Lemma 3 in combination with the ETH and the reduction from 3-CNF-Sat to 3-Dimensional Matching yields the following theorem.

##### **Theorem 1**

For any $\epsilon > 0$ with $1/\epsilon \in \mathbb {N}$, there is a $d(\epsilon )$ such that there is no $(1+\epsilon )$-approximation algorithm with running time $O\left( 2^{o\left( (1/\epsilon ) ^{d/3}\right) } (nd)^{O(1)} \right)$ for Vector Scheduling in $d\ge d(\epsilon )$ dimensions, unless the Exponential Time Hypothesis (ETH) fails.

##### *Proof*

Suppose that there exists a $(1+\epsilon )$-approximation for Vector Scheduling that runs in time $O\left( \exp \left( o((1/\epsilon )^{d/3})\right) (nd)^{O(1)}\right)$. By Lemma 3 we get an $O\left( 2^{o(q)}|T|^{O(1)}\right)$ time algorithm for 3-DM, which in turn implies an algorithm for 3-CNF-Sat that runs in time $O\left( 2^{o(M)}(N+M)^{O(1)}\right)$, which contradicts the ETH. $\square$

### Lower Bound Assuming NP has no Subexponential Time Algorithms

Lemma 3 also implies the following.

#### **Theorem 2**

For any $\epsilon > 0$ with $1/\epsilon \in \mathbb {N}$, there is a $d(\epsilon )$ such that there is no $(1+\epsilon )$-approximation algorithm with running time $O\left( 2^{(1/\epsilon )^{o(d)}} (nd)^{O(1)}\right)$ for Vector Scheduling in $d\ge d(\epsilon )$ dimensions, unless NP has subexponential time algorithms, i.e. NP $\subseteq \cap _{\epsilon >0} \text {DTIME}(2^{n^\epsilon })$.

#### *Proof*

Any problem in NP of size *n* can be reduced to an NP-complete problem of size $n^{O(1)}$ in polynomial time. In particular, any problem $\mathcal {P}$ in NP can be formulated as a 3-DM problem with at most $n^{c}$ elements and triples, for some constant *c*.

Suppose, by contradiction, that there is a $(1+\epsilon )$-approximation for Vector Scheduling that runs in time $O\left( \exp \left( (1/\epsilon )^{o(d)}\right) (nd)^{O(1)}\right)$. Setting $d = \log _{(1/\epsilon )}(n^c) + 7 \le c \log _{(1/\epsilon )} n + 7$ gives an $O\left( \exp \left( (1/\epsilon )^{o(\log _{(1/\epsilon )}(n))}\right) n^{O(1)}\right) = O\left( \exp \left( n^{o(1)}\right) \right)$ time algorithm for $\mathcal {P}$, which is subexponential. $\square$

### Lower Bound with Resource Augmentation

In this subsection we show a lower bound on the running time of $(1+\epsilon )$-approximation algorithms for Vector Scheduling that are allowed *resource augmentation*, i.e. besides exceeding the optimal load by a factor $(1+\epsilon )$, it is also allowed to use $\epsilon m$ extra machines.

To show this, we reduce from a stricter version of 3-Dimensional Matching, namely 3-Dimensional Matching- *B* , abbreviated as 3-DM- *B* . In this problem we are given a set of triples $T \subseteq X \times Y \times Z$, where *X*, *Y* and *Z* are disjoint finite sets and each element in $X \cup Y \cup Z$ appears at most *B* times in the triples of *T*. The goal is to find a subset of triples $T'$ that maximizes the number of elements in $X \cup Y \cup Z$ that appear exactly once in $T'$.

#### **Theorem 7**

(Petrank [19]) For 3-Dimensional Matching-3 it is NP-hard to distinguish between instances where all elements can be covered by disjoint triples and those instances where at most a $(1-\epsilon _{\textsc {3-DM}})$ fraction of the elements can be covered by disjoint triples, where $\epsilon _{\textsc {3-DM}} < 1$ is some universal constant.

Using this result we prove the following lemma.

#### **Lemma 4**

For Vector Scheduling in $d \ge d_0$ dimensions it is NP-hard to distinguish between instances where all jobs can be scheduled on *m* machines with maximum load 1 and those instances where all jobs can be scheduled on $(1+\epsilon _0)m$ machines with maximum load $1+\epsilon _0$, where $0 < \epsilon _0 < 1$ and $d_0 \ge 1$ are some universal constants and $1/\epsilon _0$ is integer.

#### *Proof*

Construct a Vector Scheduling instance from the 3-DM-3 problem in almost the same way as for 3-DM. The only difference is that for every (real or dummy) *X*-job *i* and triple (*i*, *j*, *k*), instead of only encoding $\langle i\rangle$ (respectively $\overline{\langle i\rangle }$), we append this by encoding $\overline{\langle i\rangle }$ (respectively $\langle i\rangle$) (all other jobs get extra zero-entries). See Table 2. Consequently, if a triple (*i*, *j*, *k*) is scheduled on a machine where also an *X*-job *x* is scheduled, then $i=x$. Previously we established this through the fact that the load in each coordinate is exactly *b*. However, here we do not have this property because of the extra machines.

**Table 2:** New construction of the *X*-jobs and triple-jobs of the 3-DM-3 problem

Job name	Values of the coordinates				
	*T* / *X* / *Y* / *Z*	Real/dummy	Encoding of element(s)		
Real *X*-job *i*:	0, *b*, 0, 0	*b*, 0, 0	$\langle i\rangle _1,\ldots ,\langle i\rangle _\ell ,\overline{\langle i\rangle }_1,\ldots ,\overline{\langle i\rangle }_\ell$	$0,\ldots ,0$	$0,\ldots ,0$
Dummy *X*-job *i*:	0, *b*, 0, 0	0, *b*, 0	$\langle i\rangle _1,\ldots ,\langle i\rangle _\ell ,\overline{\langle i\rangle }_1,\ldots ,\overline{\langle i\rangle }_\ell$	$0,\ldots ,0$	$0,\ldots ,0$
Triple (*i*, *j*, *k*):	*b*, 0, 0, 0	0, 0, 0	$\overline{\langle i\rangle }_1,\ldots ,\overline{\langle i\rangle }_\ell ,\langle i\rangle _1,\ldots ,\langle i\rangle _\ell$	$\overline{\langle j\rangle }$	$\overline{\langle k\rangle }$

There are at most 3*q* triples, where $q = |X| =|Y| = |Z|$. One direction is clear, if all 3*q* elements can be covered by disjoint triples then there is a schedule of height at most *b* on $m = 3q$ machines. For the other direction, suppose we found a $(1+\epsilon )$-approximate solution with $3q \epsilon$ extra machines. Using the same reasoning as before, we now have the following properties:

- The maximum load is *b*;
- On each machine there is at most one triple, one *X*-, one *Y*-, and one *Z*-job;
- On each machine, if there are three element-jobs, then all three are *real* jobs or all three are *dummy* jobs;
- If a triple (*i*, *j*, *k*) and an *X*-job *x*, *Y*-job *y* and *Z*-job *z* are scheduled on the same machine, then $i=x$, $j=y$ and $k=z$.

Therefore, every machine on which a triple and three real elements are scheduled, corresponds to a triple in the solution to the 3-DM-3 problem.

We will now show that there is a universal constant such that it is hard to distinguish between instances where everything fits on *m* machines with maximum load 1 and instances where everything fits on $(1+\epsilon )m$ machines with maximum load $1+\epsilon$. Consider the $3q \epsilon$ machines without a triple. These $3q \epsilon$ machines contain at most $9q \epsilon$ element-jobs. Considering that there are 3*q* machines on which $9q - 9q \epsilon$ element-jobs must be scheduled together with triples, there are at most $9q \epsilon$ machines *with* a triple but with at most two elements. Hence, there are at most $9q \epsilon + 2 (9q \epsilon )$ real elements that are scheduled on either a machine without a triple, or with a triple but with only one other element. Therefore, at least $3q - 27q \epsilon$ real elements are scheduled together with triples, which corresponds to $q - 9q \epsilon$ disjoint triples that cover $3q - 27q \epsilon$ elements. If $27\epsilon < \epsilon _{\textsc {3-DM}}$, we found a solution where more than a $(1-\epsilon _{\textsc {3-DM}})$-fraction of the elements are covered in the 3-DM-3 instance, which is NP-hard. $\square$

Following the proof of Theorem 2, this immediately implies the following.

#### **Theorem 3**

For any $\epsilon < \epsilon _0$ with $1/\epsilon \in \mathbb {N}$, there is a $d(\epsilon )$ such that there is no $(1+\epsilon )$-approximation algorithm with running time $O\left( 2^{(1/\epsilon )^{o(d)}} (nd)^{O(1)}\right)$ for Vector Scheduling in $d\ge d(\epsilon )$ dimensions, even with $\lceil \epsilon m \rceil$ extra machines, unless NP has subexponential time algorithms, i.e. NP $\subseteq \cap _{\epsilon >0} \text {DTIME}(2^{n^\epsilon })$, where $\epsilon _0 < 1$ is a universal constant. Assuming the ETH, no such algorithm can run in time $O\left( 2^{o\left( (1/\epsilon )^{d/6}\right) } (nd)^{O(1)}\right)$.

## Linear Time Approximation Algorithm

In this section we describe our linear time algorithm. Roughly, it works as follows. First, we preprocess the instance such that there are relatively few different types of large jobs at the cost of a small factor in the approximation guarantee. Next, we formulate and solve a mixed-integer linear program from which we obtain a multiset of configurations of large jobs, each of which can fit on one machine. We assign each configuration to a distinct machine, thereby assigning large jobs integrally to machines and small jobs fractionally. In the randomized algorithm, we assign the small jobs according to the probabilities obtained from the $\mathsf {MILP}$ and redistribute the small jobs on the overloaded machines over the other machines in such a way that no machine is overloaded. In the deterministic algorithm, we derandomize this step by assigning the small jobs integrally to machines in a greedy manner guided by a potential function that tracks the aggregate overload on the machines. Finally, we distribute this overload evenly over all machines ensuring the final loads of all machines is at most $1+\epsilon$.
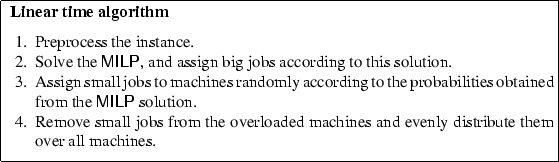


### Preprocessing

The preprocessing uses the same ideas used before in the design of approximation schemes. Typically, it is much easier to work with a few distinct jobs as we will see in the formulation of our mixed-integer linear program.
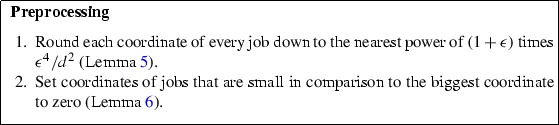


The first step is to round all coordinates of each job down to the nearest power of $(1+\epsilon )$ times a small polynomial in $\epsilon$ and 1 / *d*.

#### **Lemma 5**

([5]) Given a set *V* of jobs and $\epsilon > 0$, let *W* be a modified (multi)set of *V* where we replace each job $\mathbf {v}$ in *V* with a job $\mathbf {w}$ as follows:

$$\begin{aligned} w_j:= {\left\{ \begin{array}{ll} \epsilon ^4/d^2 \cdot (1+\epsilon )^k &{} \text { if } \exists k \in \mathbb {N}: \ \epsilon ^4/d^2 \cdot (1+\epsilon )^{k} \le v_j < \epsilon ^4/d^2 \cdot (1+\epsilon )^{k+1}, \\ 0 &{} \text { otherwise.} \end{array}\right. } \end{aligned}$$

Then, for any subset of jobs $V'\subseteq V$ with corresponding subset $W' \subseteq W$, we have $\sum _{\mathbf {v} \in V'} \mathbf {v} \le (1+\epsilon ) \sum _{\mathbf {w} \in W'} \mathbf {w}$.

Next, we ensure that the non-zero values of coordinates of a job are not too small compared to the largest coordinate of a job.

#### **Lemma 6**

([5]) Given a set *V* of jobs and $\eta > 0$, let *W* be a modified (multi)set of *V* where we replace each job $\mathbf {v}$ in *V* with a job $\mathbf {w}$ as follows:

$$\begin{aligned} w_j := {\left\{ \begin{array}{ll} 0 &{} \text {if } v_j < \eta \left\| \mathbf {v} \right\| _{\infty }, \\ v_j &{} \text {otherwise.} \end{array}\right. } \end{aligned}$$

Then, for any subset of jobs $V'\subseteq V$ with corresponding subset $W' \subseteq W$, we have $\sum _{\mathbf {v} \in V'} \mathbf {v} \le \sum _{\mathbf {w} \in W'} \mathbf {w} + \left( \eta \sum _{w \in W'} \Vert \mathbf {w}\Vert _\infty \right) \mathbf {1}$.

The following lemma states that the error due to the preprocessing of any schedule is small, and follows from the previous lemmata, setting $\eta := \epsilon /d$.

#### **Lemma 7**

Let $\epsilon > 0$, let *V* be the original set of jobs and *W* be the (multi)set of jobs preprocessed by Lemmata 5 and 6. Then for any $\mathbf {w} \in W$ and coordinate $j \in [d]$,

- if $w_j \ne 0$ then there exists a $k \in \mathbb {N}$ such that $w_j = \epsilon ^4/d^2 \cdot (1+\epsilon )^k$,
- if $$w_j \ne 0$$ then $$w_j/\Vert w\Vert _\infty \ge \epsilon / d$$.

Moreover, for any subset of jobs $V' \subset V$ such that $\sum _{\mathbf {v}\in V'} \mathbf {v} \le \mathbf {1}$ with corresponding modified subset $W' \subseteq W$, we have $\sum _{\mathbf {w} \in W'} \mathbf {w} \le \sum _{\mathbf {v} \in V'} \mathbf {v} \le (1+\epsilon ) \sum _{\mathbf {w} \in W'} \mathbf {w} + \epsilon \mathbf {1}$.

From now on, by job we mean the job preprocessed by Lemma 7.

### The Mixed-Integer Linear Program

In this subsection we describe our mixed-integer linear program and how to solve it fast. We distinguish between *small* and *big* jobs and treat them differently. A job $\mathbf {p}$ is *small* if $\left\| \mathbf {p} \right\| _\infty < \epsilon ^3/d$ and otherwise the job is *big*.

As all non-zero coordinates are at most a factor $d / \epsilon$ apart by Lemma 7, the smallest possible coordinate of any big job is $\epsilon ^4 / d^2$. Let $\mathcal {T}_{\text {big}}$ be the set of all *types* of big jobs, $\mathcal {T}_{\text {big}} := \{0,\epsilon ^4/d^2, (1+\epsilon )\epsilon ^4/d^2, (1+\epsilon )^2\epsilon ^4/d^2, \ldots , 1\}^d$. A big job $\mathbf {p}$ has type $\mathbf {t} \in \mathcal {T}_{\text {big}}$ if and only if $\mathbf {p} = \mathbf {t}$. Every big job has a corresponding type, since the rounding procedure rounded these jobs to exactly these values.

Similarly, we define a set $\mathcal {T}_{\text {small}}$ of all types of small jobs. We define the type of a small job based on its relative size in each coordinate, that is, a small job $\mathbf {p}$ has type $\mathbf {t} = (t_1, \ldots , t_d) \in \mathcal {T}_{\text {small}}$ if and only if $p_j / \left\| \mathbf {p}\right\| _\infty = t_j$ for all coordinates $j \in [d]$. As the smallest non-zero coordinate in $\mathbf {p} / \left\| \mathbf {p}\right\| _\infty$ is at least $\epsilon /d$, we define $\mathcal {T}_{\text {small}} := \{0, (1+\epsilon )^{-\ell }, (1+\epsilon )^{-\ell +1},\ldots ,(1+\epsilon )^{-1},1\}^d$, where $\ell := \left\lceil \log _{(1+\epsilon )}(d / \epsilon )\right\rceil$ is such that $(1+\epsilon )^{-\ell }$ is the smallest power of $1+\epsilon$ that is at least $\epsilon /d$. Note that each small job has exactly one type in $\mathcal {T}_{\text {small}}$ and that there are at most $T := \left\lceil 4 \log _{(1+\epsilon )}(d/\epsilon )+2\right\rceil ^d$ types of big and small jobs.

The mixed-integer linear programming has a variable for every configuration, which is a collection of big jobs together with available space for small jobs. We will call the (rounded) space for small jobs a *profile*, which is a vector from $\mathcal {F}:= \{0,\epsilon ,(1+\epsilon ) \epsilon , (1+\epsilon )^2 \epsilon ,\ldots ,1\}^d$. A *configuration* *C* is a tuple $C=(B,\mathbf {f})$, where *B* is a multiset of rounded processing times of big jobs and $\mathbf {f}$ is a profile for small jobs such that the big jobs and the profile fit together on one machine, exceeding the maximum load by only a little, i.e. $\left( \sum _{\mathbf {p} \in B} p_j\right) + f_j \le (1+\epsilon )$ for all coordinates *j*. As each big job has a coordinate of at least $\epsilon ^3 / d$, there can be no more than $d^2 / \epsilon ^3$ big jobs on a machine. As there are at most *T* types of big jobs, we know that there are at most $N \le T^{\left\lceil d^2/\epsilon ^3\right\rceil } \cdot T$ different configurations.

We now describe our mixed-integer linear program. Let $\mathcal {C}$ be the set of all configurations and let $x_{C}$ denote the number of machines that have jobs assigned to them according to configuration $C \in \mathcal {C}$. Let $n(C,\mathbf {t})$ denote the number of big jobs of type $\mathbf {t}$ in configuration *C*, and let $n(\mathbf {t})$ denote the total number of big jobs of type $\mathbf {t}$ in the instance. Denote the set of small jobs of type $\mathbf {t}$ assigned to configurations having profile $\mathbf {f}$ by $J(\mathbf {f},\mathbf {t})$, and let the variables $y_{\mathbf {f},\mathbf {t}} = \sum _{p \in J(\mathbf {f},\mathbf {t})} \Vert p\Vert _\infty$ denote the sum of their largest coordinates, their *amount*. Let $a(\mathbf {t}) := \sum _{\mathbf {p} :\mathbf {p} \text { is of small type } \mathbf {t}} \left\| \mathbf {p} \right\| _\infty$ denote the total amount of small jobs of type $\mathbf {t}$ in the instance. Consider the following program.
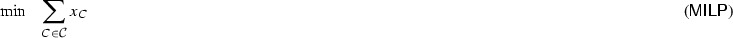

C1 $$\begin{aligned} \text {s.t.} \quad&\sum _{C \in \mathcal {C}} x_{C} \cdot n(C,\mathbf {t}) \ge n(\mathbf {t})&\quad \forall \mathbf {t} \in \mathcal {T}_{\text {big}}\qquad \qquad \qquad \qquad \qquad \end{aligned}$$

C2 $$\begin{aligned}&\sum _{\mathbf {f} \in \mathcal {F}} y_{\mathbf {f},\mathbf {t}} \ge a(\mathbf {t})&\forall \mathbf {t} \in \mathcal {T}_{\text {small}}\quad \qquad \qquad \qquad \qquad \qquad \qquad \end{aligned}$$

C3 $$\begin{aligned}&\sum _{\mathbf {t} \in \mathcal {T}_{\text {small}}} y_{\mathbf {f},\mathbf {t}} \cdot t_i/\left\| \mathbf {t}\right\| _{\infty } \le f_i \cdot \sum _{C : C=(B,\mathbf {f})} x_{C} \quad \forall i \in [d], \mathbf {f} \in \mathcal {F}\\&\mathbf {x} \in \mathbb {Z}^{\mathcal {C}}&\nonumber \\&\mathbf {y},\mathbf {x} \ge 0&\nonumber \end{aligned}$$

The first and second constraint ensure that the big and the small jobs are covered integrally (respectively fractionally). The third constraint ensures that small jobs fit in the machine profiles, as it requires that for each profile *f*, the cumulative amount of small jobs of type *t* that are assigned to *f* is at most the total amount of *f*. These are valid constraints for any feasible solution.

#### **Lemma 8**

An optimal solution to $\mathsf {MILP}$ can be found in time $O\left( \exp \left( {(1/\epsilon )^{O(d \log \log d)}}\right) \right. \left. \cdot \log (nd) \right) .$

#### *Proof*

First, we bound the number of choices for non-zero integer variables. To do that, suppose that there is a finite solution and suppose that the continuous variables $y_{\mathbf {f},\mathbf {t}}$ are fixed: this allows us to disregard constraints (C2), only containing continuous variables. Then introduce slack variables such that all constraints are equality constraints and the $\mathsf {MILP}$ matches the form of Theorem 6. For the application of this theorem we can disregard the non-negativity constraints [6]. Thus, we are left with at most $|\mathcal {T}_{\text {big}}|+d|\mathcal {F}| \le (d+1) T$ constraints. The largest size of the coefficients are the constants $n(C,\mathbf {t})$, $t_i$, $\left\| \mathbf {t}\right\| _\infty$ and $f_i$, all of which require at most $d^2/\epsilon ^3$ bits to describe. By Theorem 6 there is an optimal solution such that there are at most $2 \left( (d+1)T+1 \right) \left( \log \left( (d+1)T+1 \right) + d^2/\epsilon ^3 + 2 \right)$ non-zero integer variables. As

$$\begin{aligned} \log ((d+1)T+1)&= \log \left( (d+1) \left\lceil 4 \log _{(1+\epsilon )} (d/\epsilon ) +2 \right\rceil ^d + 1 \right) \\&\le d \log \left( 4 \log _{(1+\epsilon )} (d/\epsilon ) \right) \le d^2 / \epsilon ^3, \end{aligned}$$

the number of non-zero integer variables is at most

$$\begin{aligned} 2 \left( (d+1)T+1 \right) \left( 2 d^2 / \epsilon ^3 + 2 \right)&= 4 \left( \left( (d^3+d^2)/\epsilon ^3 + d + 1 \right) T + d^2/\epsilon ^3 + 1 \right) \\&\le 8 \left( \left( d^3/\epsilon ^3 + d \right) T + d^2/\epsilon ^3 \right) \le 16 T d^3/\epsilon ^3. \end{aligned}$$

Therefore, we can bound the number of choices for non-zero variables by

$$\begin{aligned} N^{16Td^3/\epsilon ^3} \le \left( T^{d^2/\epsilon ^3 + 2}\right) ^{16Td^3/\epsilon ^3} = 2^{(d^2/\epsilon ^3+2) 16T\log T d^3/\epsilon ^3}. \end{aligned}$$

Using that $T \log T \le T^2$ and plugging in the definition of *T*, we bound this by

$$\begin{aligned} 2^{(16 d^6/\epsilon ^6) T^2} = \exp \left( 16d^6/\epsilon ^6 \left\lceil 3 \log _{(1+\epsilon )}(d/\epsilon )+2\right\rceil ^{2d} \right) . \end{aligned}$$

As the first part is $(1/\epsilon )^{O(\log (d))}$ and the second part is $(1/\epsilon )^{O(d \log \log d)}$, the number of choices for non-zero variables is at most $\exp \left( (1/\epsilon )^{O(d \log \log d)}\right)$.

Now we ‘guess’ the non-zero variables by enumerating all possible choices, and solve the $\mathsf {MILP}$ for only those variables. Since there are at most $16T d^3/\epsilon ^3$ variables, by Theorem 5 solving the $\mathsf {MILP}$ takes time $O\left( (16T d^3/\epsilon ^3)^{40T d^3/\epsilon ^3} \cdot s\right)$, where *s* denotes the maximum length of the binary encoding of the mixed-integer linear program. Using the same rewriting as above, we can rewrite this to $O\left( \text {exp}\left( {(1/\epsilon )^{O(d \log \log d)}}\right) \cdot s\right)$. As the mixed-integer linear program can be described using $s = TN \log (nd)$ bits, the proof is complete. $\square$

### Randomized Algorithm

In this subsection we sketch step 3 and 4 of the algorithm, the integral assignment of small jobs to machines using the solution to $\mathsf {MILP}$.

For step 3, recall that $y_{\mathbf {f},\mathbf {t}}$ is the amount of small jobs of type $\mathbf {t}$ that are assigned to profile $\mathbf {f}$. For each small job type $\mathbf {t}$, let $\beta (\mathbf {f},\mathbf {t})$ denote the fraction of type $\mathbf {t}$ assigned to profile $\mathbf {f}$:

$$\begin{aligned} \beta (\mathbf {f},\mathbf {t}) := \frac{y_{\mathbf {f},\mathbf {t}}}{\sum _{\mathbf {g} \in \mathcal {F}} y_{\mathbf {g},\mathbf {t}}}. \end{aligned}$$

For each small job $\mathbf {p}$ of type $\mathbf {t}$, pick a profile $\mathbf {f}$ randomly with probability $\beta _{\mathbf {f},\mathbf {t}}$ and then pick a machine uniformly at random among the ones with profile $\mathbf {f}$. Assign job $\mathbf {p}$ to this machine.

For step 4, we call a machine with profile $\mathbf {f}$ *overloaded* if the load of small jobs exceeds $\mathbf {f} + \epsilon \cdot \mathbf {1}$ in some coordinate. We take all the small jobs on overloaded machines and distribute them among all machines using a linear time simple sequential assignment. We will prove that the probability that the load on a machine in a coordinate exceeds the profile by more than $\epsilon$ is exponentially small. This implies that the expected overload on each machine is small, hence, the total overload over all the machines is small.

For the following proofs we fix a machine. Define for each small job $\mathbf {p}$ and coordinate *j* the random variables $X_{\mathbf {p}}^j$ with $\mu _{\mathbf {p}}^j$ as its mean, which is the contribution of job $\mathbf {p}$ to the *j*-th coordinate of the machine:

$$\begin{aligned} X_{\mathbf {p}}^j = \left\{ \begin{array}{ll} p_j, &{} \quad \hbox {if job }\mathbf {p} \hbox { is assigned to the machine;} \\ 0, &{} \quad \hbox {otherwise.} \end{array} \right. \end{aligned}$$

For a coordinate *j*, let $X_j := \sum _{\text {small job } \mathbf {p}} \, X_{\mathbf {p}}^j$ denote the load of small jobs on the machine. We need the following Bernstein’s inequality.

#### **Theorem 8**

Let $X_1,\ldots ,X_n$ be independent random variables with $E[X_i]=\mu _i$ and $|X_i - \mu _i| \le M$ for all *i*. Let $\mu = \sum _i E[X_i]$ and $\sigma _i^2 = E[(X_i-\mu _i)^2]$. Then for any $t > 0$, it holds that

$$\begin{aligned} Pr\left( \sum _i X_i > \mu + t\right) \le \exp \left( -\frac{t^2/2}{\left( \sum _i \sigma _i^2\right) + Mt/3}\right) . \end{aligned}$$

We first show that the probability that the load of small jobs exceeds the profile in a coordinate is small.

#### **Lemma 9**

For any machine with profile $\mathbf {f}$ and $0 < \epsilon < 1$ we have

$$\begin{aligned} Pr[X_j \ge f_j + \epsilon ] \le e^{-\epsilon ^2/4\delta } \text { for all coordinates } j \in [d], \end{aligned}$$

where $\delta$ is the maximum coordinate of any small job.

#### *Proof*

Let $m(\mathbf {f})$ denote the number of machines with profile $\mathbf {f}$. Since a job $\mathbf {p}$ of type $\mathbf {t}$ is assigned to this machine with probability $\beta (\mathbf {f},\mathbf {t})/m(\mathbf {f})$, the expected load on coordinate *j* on that machine is

$$\begin{aligned} \sum _{\mathbf {t} \in \mathcal {T}_{\text {small}}} \sum _{\mathbf {p}: \mathbf {p} \text { of type } \mathbf {t}} p_j \frac{\beta (\mathbf {f},\mathbf {t})}{m(\mathbf {f})} = \sum _{\mathbf {t} \in \mathcal {T}_{\text {small}}} \sum _{\mathbf {p}: \mathbf {p} \text { of type } \mathbf {t}} p_j \frac{ y_{\mathbf {f},\mathbf {t}}}{m({\mathbf {f}}) (\sum _{\mathbf {g} \in \mathcal {F}} y_{\mathbf {g},\mathbf {t}})}. \end{aligned}$$

Recall that $f_j$ is the space available for small jobs in coordinate *j* of profile $\mathbf {f}$ and that $a(\mathbf {t})$ is the amount of small jobs of type $\mathbf {t}$. Therefore, $\sum _{\mathbf {p}: \mathbf {p} \text { of type } \mathbf {t}} p_j = a(\mathbf {t}) t_j/\Vert \mathbf {t}\Vert _\infty$, and hence the expected load is at most

$$\begin{aligned} \sum _{\mathbf {t}\in \mathcal {T}_{\text {small}}} \frac{a(\mathbf {t}) y_{\mathbf {f},\mathbf {t}} t_j}{ \Vert \mathbf {t}\Vert _\infty m({\mathbf {f}}) \sum _{\mathbf {g} \in \mathcal {F}} y_{\mathbf {g},\mathbf {t}}} \le \sum _{\mathbf {t}\in \mathcal {T}_{\text {small}}} \frac{t_j y_{\mathbf {f},\mathbf {t}}}{ \Vert \mathbf {t}\Vert _\infty m(\mathbf {f})} \le f_j. \end{aligned}$$

Both inequalities follow from the $\mathsf {MILP}$ constraints: the first follows as $\sum _{\mathbf {f} \in \mathcal {F}} y_{\mathbf {f},\mathbf {t}} \ge a(\mathbf {t})$ and the second follows as $\sum _{\mathbf {t}\in \mathcal {T}_{\text {small}}} y_{\mathbf {f},\mathbf {t}} t_j/\Vert \mathbf {t}\Vert _\infty \le m(\mathbf {f}) f_j$.

We now apply Bernstein’s inequality to our setting. We have that

$$\begin{aligned} (\sigma _{\mathbf {p}}^j)^2&:= E[(X_{\mathbf {p}}^j - \mu _{\mathbf {p}}^j)^2] \\&= \left( p_j - p_j \frac{\beta (\mathbf {f},\mathbf {t})}{m(\mathbf {f})} \right) ^2 \frac{\beta (\mathbf {f},\mathbf {t})}{m(\mathbf {f})} + \left( 0 - p_j \frac{\beta (\mathbf {f},\mathbf {t})}{m(\mathbf {f})} \right) ^2 \left( 1 - \frac{\beta (\mathbf {f},\mathbf {t})}{m(\mathbf {f})} \right) \\&= p_j^2 \left( 1 - \frac{\beta (\mathbf {f},\mathbf {t})}{m(\mathbf {f})} \right) \frac{\beta (\mathbf {f},\mathbf {t})}{m(\mathbf {f})} \le (p_j)^2 \frac{\beta (\mathbf {f},\mathbf {t})}{m_{\mathbf {f}}}. \end{aligned}$$

Thus,

$$\begin{aligned} \sum _{\text {small job } \mathbf {p}} (\sigma _{\mathbf {p}}^j)^2 \le \left( \max _{\text {small job } \mathbf {p} } p_j \right) \sum _{\text {small job } \mathbf {p}} \frac{\beta (\mathbf {f},\mathbf {t})}{m(\mathbf {f})} p_j \le \delta f_j \le \delta . \end{aligned}$$

Moreover $|X_{\mathbf {p}}^j - \mu _{\mathbf {p}}^j| \le \delta$, so choose $M = \delta$. Then

$$\begin{aligned} Pr\left[ X_j > f_j + x\right] \le \exp \left( \frac{-x^2/2}{ \delta + \delta x/3}\right) . \end{aligned}$$

For $x \le 3$, we bound this by $\exp (-x^2/(4\delta ))$. For $x\ge 3$, we bound this by

$$\begin{aligned} \exp \left( -\frac{x^2/2}{2\delta x/3}\right) = \exp (-3x/4\delta ). \end{aligned}$$

So, for any $\epsilon \le 1$, $Pr[X_j \ge f_j + \epsilon ] \le e^{-\epsilon ^2/4 \delta }$. $\square$

We now bound $E[X_j|X_j \ge f_j + \epsilon ] Pr[X_j \ge f_j + \epsilon ]$, i.e. the average load on overloaded machines.

#### **Lemma 10**

For any machine with profile $\mathbf {f}$ and $0 < \epsilon < 1/5$ we have

$$\begin{aligned} E[X_j | X_j \ge f_j + \epsilon ] Pr[X_j \ge f_j + \epsilon ] \le 2 \epsilon ^3/d^3 \text { for all coordinates } j. \end{aligned}$$

#### *Proof*

Recall that for any non-negative random variable *Y* with finite mean,

$$\begin{aligned} E[Y] = \int _{0}^\infty Pr[Y \ge y] dy. \end{aligned}$$

This implies that

$$\begin{aligned} E[Y|(Y >t)] = t + \frac{1}{Pr[Y\ge t]} \int _{y=0}^\infty Pr[Y \ge t+y] dy. \end{aligned}$$

Applying this to our setting, we get

$$\begin{aligned} E[X_j|X_j\ge & {} (f_j + \epsilon )] Pr[X_j \ge f_j + \epsilon ] \\\le & {} (f_j + \epsilon ) Pr[X_j \ge f_j + \epsilon ] + \int _{x=0}^\infty Pr[X_j> f_i + \epsilon + x] dx. \end{aligned}$$

As $f_i\le 1$ and by the proof of Lemma 9, this is at most

1 $$\begin{aligned} (1+\epsilon ) Pr[X_j \ge f_i + \epsilon ] + \int _{\epsilon }^3 \exp (-x^2/4 \delta ) dx + \int _{3}^\infty \exp (-3x/4\delta ) dx, \end{aligned}$$

where $\delta := \max _{\mathbf {p}: \mathbf {p} \text { small job}} |\mathbf {p}\Vert _\infty$ is the maximum coordinate of any small job. The last term is $(4 \delta /3) \exp (-9/4\delta )$. The second term can be upper bounded by $\int _{\epsilon }^\infty \exp (-x^2/4 \delta ) dx$. Let $f(x) = \frac{1}{\sqrt{2\pi }} \exp (-x^2/2)$ denote the pdf of the standard gaussian *N*(0, 1). Let $\overline{\Phi }(x) = \int _x^\infty f(y) dy$. Using that $\overline{\Phi }(x) \le f(x)/x$ for any $x>0$, it follows that

$$\begin{aligned} \int _{\epsilon }^\infty \exp (-x^2/4 \delta ) dx = \sqrt{2\delta } \int _{\epsilon /\sqrt{2\delta }}^{\infty } e^{-y^2/2} dy \le (2 \delta \sqrt{2\pi }/\epsilon ) \exp (-\epsilon ^2/4\delta ). \end{aligned}$$

We plug this in Eq. (1), bounding $\delta$ by $\epsilon ^2/(4\ln (d/\epsilon ))$, which is larger than $\epsilon ^3/d$ if $d/\epsilon \ge 9$. As $d \ge 2$ and $\epsilon < 1/5$ this is a valid upper bound for all small jobs. This yields that the total expected load on overloaded machines is at most

$$\begin{aligned}&\left( 1 + \epsilon + 2\delta \sqrt{2\pi }/\epsilon \right) \exp (-\epsilon ^2/4\delta ) + (4\delta /3) \exp (-9/4\delta ) \\&\quad =\left( 1 + \epsilon + \frac{\epsilon \sqrt{2\pi }}{2\ln (d/\epsilon )} \right) \epsilon /d + \frac{\epsilon ^2}{3\ln (d/\epsilon )} (\epsilon /d)^{9/\epsilon ^2}. \end{aligned}$$

This is at most $2 \epsilon /d$. $\square$

We can now prove the following theorem.

#### **Theorem 9**

There is an algorithm that runs in $O\left( 2^{(1/\epsilon )^{O(d \log \log d)}} + nd\right)$ time and finds a schedule such that the load on each machine is at most $1+\epsilon$ with high probability.

#### *Proof*

Let $\epsilon ' := \epsilon /9$. First we prove the approximation ratio. For an overloaded machine *k*, let $L_k$ be the sum of the $\ell _1$-norm of all small jobs assigned to *k*, and let *L* be the sum of the $\ell _1$-norm of all small jobs on all overloaded machines. By Lemma 10 we know that $E[L_k] \le 2 \epsilon '$ for all machines *k* and thus, by linearity of expectation, $E[L] \le 2 m \epsilon '$. Therefore $Pr[L > 4m\epsilon '] < 1/2$ by Markov’s inequality. (One can prove the $L_k$ variables are negatively associated, and therefore by standard Chernoff-Hoeffding bounds the probability of having at least total overload *t* is exponentially small for any $t>0$.) Remove all small jobs assigned from the overloaded machines and order them arbitrarily. Greedily group them together until the $\ell _1$-norm exceeds $4\epsilon '$ and then start a new group. Every group has size at most $4 \epsilon ' + \delta$. Now assign every group to a non-overloaded machine. The small jobs on the overloaded machines have now been redistributed such that the extra load on every machine is in expectation at most the average plus the largest small job size, i.e. $4\epsilon ' + \delta \le 4\epsilon ' + \epsilon '^3/d \le 5 \epsilon '$. All other machines exceeded their profile in each coordinate by at most $\epsilon '$. Additionally, from the mixed-integer linear program we lost another $\epsilon '$ since we only required that the big jobs and the profile add up to at most $1+\epsilon '$. This gives a total of $7\epsilon '$ on the preprocessed instance and factoring in the preprocessing we get $(1+\epsilon ')(7\epsilon ')+\epsilon ' \le 9\epsilon ' = \epsilon$.

The preprocessing and randomized rounding steps can be implemented in *O*(*nd*) time. To bound the time of solving the mixed-integer linear program, we use the fact that $ab \le a^2 + b^2$. Choosing $a= 2^{(1/\epsilon ')^{O(d \log \log d)}}$ and $b = \log (nd)$, we get $O\left( 2^{(1/\epsilon ')^{O(d \log \log d)}}\log (nd) \right) \le O\left( 2^{2(1/\epsilon ')^{O(d \log \log d)}} + \log ^2(nd)\right)$, so the total running time is at most $O\left( 2^{(1/\epsilon ')^{O(d \log \log d)}} + nd\right) .$ $\square$

By simply repeating the rounding and grouping step until a solution is found, we get an *O*(*nd*) time algorithm for assigning small jobs that returns a $(1+\epsilon )$-approximation with high probability.

### Deterministic Algorithm

Recall that the $\mathsf {MILP}$ only gives an assignment of small job types to profiles, while we need an assignment of individual jobs to machines for a deterministic algorithm. This can be done in three steps using standard techniques. First, small job types are assigned integrally to profiles. Then, using a pessimistic estimator, small jobs are integrally assigned to machines having a fixed profile. Finally, a direct calculation shows that the the total load on overloaded machines is at most $O(\epsilon m/d)$, so the small jobs from these machines can be redistributed over all machines in a round-robin fashion without increasing the loads too much.

From this and Theorem 9, we have our main theorem.

#### **Theorem 4**

For any $\epsilon >0$ and $d \ge 1$, there is a deterministic $(1+\epsilon )$-approximation algorithm for *d*-dimensional Vector scheduling that runs in time $O\left( 2^{(1/\epsilon )^{O(d \log \log d)}}+ nd \right) .$

## References

[1] Alon N, Azar Y, Woeginger GJ, Yadid T (1998). Approximation schemes for scheduling on parallel machines. J. Sched..

[2] Bansal, N., Vredeveld, T., van der Zwaan, R.: Approximating vector scheduling: almost matching upper and lower bounds. In: Proceedings of 11th Latin American Symposium on Theoretical Informatics of Theoretical Computer Science and General Issues, vol. 8392, pp. 47–59. Springer, Berlin Heidelberg (2014)

[3] Bonifaci, V., Wiese, A.: Scheduling unrelated machines of few different types. CoRR, abs/1205.0974, (2012)

[4] Calabro, C., Impagliazzo, R., Paturi, R.: A duality between clause width and clause density for SAT. In IEEE conference on computational complexity, pp. 252–260. IEEE computer society, (2006)

[5] Chekuri C, Khanna S (2004). On multidimensional packing problems. Soc. Ind. Appl. Math. J. Comput..

[6] Eisenbrand F, Shmonin G (2006). Carathéodory bounds for integer cones. Oper. Res. Lett..

[7] Epstein L, Tassa T (2003). Vector assignment problems: a general framework. J. Algorithm.

[8] Epstein L, Tassa T (2006). Vector assignment schemes for asymmetric settings. Acta Inform..

[9] Frank A, Tardos E (1987). An application of simultaneous diophantine approximation in combinatorial optimization. Combinatorica.

[10] Garey MR, Johnson DS (1979). Computers and Intractability: A Guide to the Theory of NP-Completeness.

[11] Hochbaum DS, Shmoys DB (1987). Using dual approximation algorithms for scheduling problems: theoretical and practical results. J. Assoc. Comput. Mach..

[12] Hochbaum DS, Shmoys DB (1988). A polynomial approximation scheme for scheduling on uniform processors: using the dual approximation approach. Soc. Ind. Appl. Math. J. Comput..

[13] Impagliazzo R, Paturi R, Zane F (2001). Which problems have strongly exponential complexity?. J. Comput. Syst. Sci..

[14] Jansen K (2010). An EPTAS for scheduling jobs on uniform processors: using an MILP relaxation with a constant number of integral variables. Soc. Ind. Appl. Math. J. Discret. Math..

[15] Kannan R (1987). Minkowski’s convex body theorem and integer programming. Math. Oper. Res..

[16] Karmarkar, N., Karp, R.M.: An efficient approximation scheme for the one-dimensional bin-packing problem. In: Proceedings of the 23rd Annual Symposium on Foundations of Computer Science. SFCS ’82, pp. 312–320. IEEE Computer Society, Washington, DC (1982)

[17] Lenstra HW (1983). Integer programming with a fixed number of variables. Math. Oper. Res..

[18] Meyerson A, Roytman A, Tagiku B, Raghavendra P, Raskhodnikova S, Jansen K, Rolim J (2013). Online multidimensional load balancing. Approximation, Randomization, and Combinatorial Optimization.

[19] Petrank E (1994). The hardness of approximation: gap location. Comput. Complex..

[20] Rothvoss, T.: Approximating bin packing within O(log OPT * Log Log OPT) bins. In: IEEE 54th Annual Symposium on Foundations of Computer Science, pp. 20–29. IEEE, Berkeley, CA (2013)

